# Effect of Body Mass Index on the Prognostic Value of Atherogenic Index of Plasma in Patients with Acute Coronary Syndrome Undergoing Percutaneous Coronary Intervention

**DOI:** 10.3390/jcm12206543

**Published:** 2023-10-16

**Authors:** Yi Kan, Yan Sun, Hua Shen, Xiaoli Liu, Yuyang Liu, Dongmei Shi, Xiaoteng Ma, Yujie Zhou

**Affiliations:** 1 Department of Cardiology, Beijing Anzhen Hospital, Capital Medical University, Beijing 100029, China; 2 Beijing Institute of Heart Lung and Blood Vessel Disease, Beijing Key Laboratory of Precision Medicine of Coronary Atherosclerotic Disease, Clinical Center for Coronary Heart Disease, Capital Medical University, Beijing 100029, China

**Keywords:** atherogenic index of plasma, body mass index, acute coronary syndrome, percutaneous coronary intervention, adverse cardiovascular events

## Abstract

(1) Background: The aim of this study was to investigate whether the prognostic value of the atherogenic index of plasma (AIP) for adverse cardiovascular events in acute coronary syndrome (ACS) patients undergoing percutaneous coronary intervention (PCI) varied across different BMI groups. (2) Methods: This study was a retrospective analysis of a prospective registry involving 1725 ACS patients undergoing PCI. The primary endpoint was a composite of all-cause death, non-fatal ischemic stroke, non-fatal spontaneous myocardial infarction (MI), and unplanned repeat revascularization. (3) Results: The study population finally consisted of 526 patients with BMI < 24 kg/m^2^ (age 62 ± 10 years; male 64.3%), 827 patients with 24 kg/m^2^ ≤ BMI < 28 kg/m^2^ (age 60 ± 10 years; male 81.8%), and 372 patients with BMI ≥ 28 kg/m^2^ (age 57 ± 11 years; male 81.2%). The AIP as a continuous variable increased the risk for the primary endpoint in ACS patients undergoing PCI with BMI < 24 kg/m^2^ (HR 2.506; 95% CI 1.285–4.885; *p* = 0.007), while it did not increase the risk in patients with BMI ≥ 24 kg/m^2^ (hazard ratio [HR]: 1.747; 95% CI 0.921–3.316; *p* = 0.088 for patients with 24 kg/m^2^ ≤ BMI < 28 kg/m^2^; and HR: 2.096; 95% CI 0.835–5.261; *p* = 0.115 for patients with BMI ≥ 28 kg/m^2^, respectively). Compared with the lowest AIP tertile, the top AIP tertile was associated with a significantly increased risk of the primary endpoint in BMI < 24 kg/m^2^ group (HR: 1.772, 95% CI: 1.110 to 2.828, *p* = 0.016). (4) Conclusions: The AIP was significantly associated with an increased risk of adverse cardiovascular events in ACS patients undergoing PCI with BMI < 24 kg/m^2^, but not in the patients with BMI ≥ 24 kg/m^2^.

## 1. Introduction

Coronary artery disease (CAD) still has high morbidity and mortality [1,2,3] worldwide, with acute coronary syndrome (ACS) being the most severe type of CAD [4,5]. Lipid metabolism, especially elevated low-density lipoprotein-cholesterol (LDL-C) particles, plays a role in the development of ACS. Despite reaching the recommended LDL-C level, there are still quite a few patients with recurrent adverse cardiovascular events [6,7,8]. Recent studies have shown that the small dense low-density lipoprotein (sdLDL), a subfraction of LDL, may confer a superior relationship with premature atherosclerosis compared to overall LDL-C [8]. Nevertheless, the complexity and cost of measuring sdLDL limit its routine use in clinical practice [9]. Intriguingly, the atherogenic index of plasma (AIP) reflects a correlation with smaller LDL-C particles and an increased fractional esterification rate for cholesterol in plasma [10,11,12,13]. Therefore, the AIP may be useful for assessing the risk of cardiovascular disease in patients with lipid metabolism disorders.

Several studies have indicated that the AIP is strongly related to adverse cardiovascular events [14,15,16,17,18,19,20,21]. Recently, one study demonstrated that AIP could predict the incidence of stroke only in patients with a healthy body mass index (BMI) [17]. However, it remains unclear whether BMI levels are associated with the prognostic value of the AIP for adverse cardiovascular events in ACS patients. Thus, the purpose of this study was to investigate whether the prognostic value of AIP varies among ACS patients with different BMI levels.

## 2. Methods

### 2.1. Study Population

This study conducted a retrospective analysis using data from a single-center prospective registry, involving 1725 patients with Acute Coronary Syndrome who underwent either primary or elective Percutaneous Coronary Intervention (PCI) between June 2016 and November 2017.

Inclusion criteria for participants were defined as follows: individuals diagnosed with ACS in accordance with the guidelines established by the American College of Cardiology/American Heart Association [22,23], and who underwent treatment with primary or elective PCI. Exclusion criteria encompassed individuals meeting any of the following conditions: prior coronary artery bypass grafting, severe renal dysfunction, left ventricular ejection fraction (LVEF) less than 30%, cardiogenic shock, or incomplete follow-up.

ACS serves as a valuable operational term referring to a spectrum of conditions that are consistent with acute myocardial ischemia and/or infarction, typically stemming from a sudden reduction in coronary blood flow. ST-Segment Elevation Myocardial Infarction (STEMI) represents a clinical syndrome characterized by identifiable symptoms of myocardial ischemia accompanied by persistent electrocardiographic (ECG) ST elevation and subsequent release of biomarkers signifying myocardial necrosis. The absence of persistent ST-elevation suggests Non-ST-elevation ACS (NSTE-ACS), with exceptions for patients experiencing genuine posterior myocardial infarction (MI). Non-ST-elevation acute coronary syndromes can be further categorized based on cardiac biomarkers of necrosis. Elevated cardiac biomarkers in an appropriate clinical context indicate non-ST-elevation myocardial infarction (NSTEMI), while patients without these markers are classified as having unstable angina (UA).

Body Mass Index was calculated using the formula weight (kg)/height^2^ (m^2^). Participants were classified into four categories based on Chinese criteria: underweight (<18.5 kg/m^2^), normal weight (18.5 to 23.9 kg/m^2^), overweight (24.0 to 27.9 kg/m^2^), or obese (≥28.0 kg/m^2^). Cardiogenic shock was defined as a condition characterized by persistent systolic blood pressure below 90 mmHg despite adequate blood perfusion, accompanied by clinical manifestations or laboratory findings indicative of hypoperfusion. Alternatively, it was defined as a state where systolic blood pressure was maintained above 90 mmHg through the use of positive inotropic agents and/or mechanical circulatory support. Severe renal dysfunction was defined as a glomerular filtration rate (GFR) below 30 mL/min/1.73 m^2^.

None of the patients received medications intended specifically for elevating high-density lipoprotein cholesterol (HDL-C) or reducing triglyceride levels, such as niacin, fibrates, or omega-3 fatty acids, either before admission or at the time of discharge. Regarding statins, all patients were prescribed either atorvastatin or rosuvastatin, with the majority adhering to regular doses, namely atorvastatin 20 mg or rosuvastatin 10 mg.

### 2.2. Measurement

We collected demographic and medical history information from all patients through standardized questionnaires. BMI was computed as weight (kg) divided by height squared (m^2^). The central laboratory at Beijing Anzhen Hospital conducted measurements of fasting plasma glucose (FPG), total cholesterol, HDL-C, and triglyceride levels post-admission. We employed the Friedewald equation to determine LDL-C levels. Dyslipidemia was diagnosed when fasting total cholesterol exceeded 5.17 mmol/L, LDL-C surpassed 3.36 mmol/L, triglycerides were above 1.69 mmol/L, HDL-C was less than 1.03 mmol/L, or if patients were on chronic lipid-lowering medication. Additionally, AIP was calculated as the logarithm base 10 of the ratio between triglyceride and HDL-C plasma concentrations [24].

### 2.3. Follow-Up and Endpoints

The follow-up assessments were scheduled at 1 month post-hospital discharge and subsequently every 6 months. Trained personnel, unaware of patients’ baseline data, collected information on adverse events by conducting telephone interviews with patients or their family members using standardized questionnaires. The identification of adverse events was based on a meticulous review of corresponding medical records. The primary endpoint encompassed all-cause mortality, non-fatal ischemic stroke, non-fatal spontaneous myocardial infarction, and unplanned repeat revascularization. The secondary endpoint comprised each individual component event of the primary endpoint. The follow-up period extended until November 2019.

### 2.4. Statistical Analysis

The patients were categorized into 4 groups based on BMI: underweight (<18.5 kg/m^2^), normal weight (18.5 to 23.9 kg/m^2^), overweight (24.0 to 27.9 kg/m^2^) and obesity (≥28.0 kg/m^2^). Since there were only five patients in the underweight group, they were combined with the normal weight group. Patients with a BMI < 24 kg/m^2^ were stratified into three groups (T1 [AIP ≤ −0.0458], T2 [−0.0458 < AIP ≤ 0.2262], T3 [AIP > 0.2262]) based on AIP tertiles. Categorical variables were presented as frequencies and percentages. We assessed the statistical significance of differences in categorical variables between groups using either the chi-squared test or Fisher’s exact test. For continuous variables following parametric distributions, we presented them as means along with standard deviations. In cases where continuous variables exhibited non-parametric distributions, we reported medians along with interquartile ranges. To gauge the statistical significance of disparities in continuous variables across groups, we employed either the unpaired *t*-test or Mann–Whitney U test for two groups and analysis of variance or Kruskal–Wallis H test for multiple groups. For survival analyses concerning both primary and secondary endpoints, we conducted Kaplan–Meier curve analyses and employed Cox proportional hazards models.

The log-rank test was utilized to assess differences between Kaplan–Meier estimates. The results of the Cox proportional hazards analysis were presented as hazard ratios (HR) accompanied by 95% confidence intervals (CI). In the multivariate Cox proportional hazards model, we included variables that demonstrated statistical significance in the univariate analysis. For the primary endpoint, the multivariate model incorporated the following confounding variables: male (yes or no), age (continuous, per 1-year increase), current smoking (yes or no), hypertension (with or without), diabetes (with or without), cardiac failure (with or without), NSTE-ACS (with or without), CKD (with or without), previous MI (with or without), past PCI (with or without), LDL-C (continuous, per 1-unit increase), hs-CRP (continuous, per 1-unit increase), SYNTAX score (score < 22 as reference), and complete revascularization (yes or no). Statistical analyses were performed using SPSS version 27.0. A two-sided *p*-value < 0.05 was considered statistically significant.

## 3. Results

A total of 1725 ACS patients who underwent PCI were analyzed. Among them, 526 patients had a BMI < 24 kg/m^2^ (mean age 62 ± 10 years; male 64.3%), 827 patients had a BMI between 24 kg/m^2^ and 28 kg/m^2^ (mean age 60 ± 10 years; male 81.8%), and 372 patients had a BMI ≥ 28 kg/m^2^ (mean age 57 ± 11 years; male 81.2%).

Table 1 presents the baseline clinical and laboratory characteristics of the study population categorized by BMI and AIP tertiles. Patients with higher AIP values tended to be younger, had a higher prevalence of chronic daily drinking and dyslipidemia, but a lower likelihood of being diagnosed with unstable angina pectoris. Furthermore, patients with higher AIP values exhibited elevated levels of total cholesterol, LDL-C, triglycerides, and Hs-CRP, while experiencing lower levels of HDL-C.

Table 2 provides a summary of medication usage, angiographic findings, and procedural outcomes for the study population. The use of medications at discharge, angiographic findings, and procedural results were found to be similar across the various AIP groups.

During the follow-up period, a primary outcome event was confirmed in 357 patients—128 in the BMI < 24 kg/m^2^ group, 144 in the 24 kg/m^2^ ≤ BMI < 28 kg/m^2^ group, and 85 in the BMI ≥ 28 kg/m^2^ group. Among the 128 patients who experienced at least one adverse cardiovascular event in the BMI < 24 kg/m^2^ group, 29 (16.6%) were from the T1 group, 41 (23.3%) from the T2 group, and 58 (33.1%) from the T3 group. Among these 128 patients, 24 deaths occurred (21 due to cardiovascular causes and 3 due to non-cardiovascular causes), 17 experienced non-fatal spontaneous myocardial infarctions, 10 had non-fatal ischemic strokes, and 99 underwent unplanned repeat revascularization.

Table 3 presents the results of univariate and multivariate Cox proportional hazards analyses for the primary endpoint during the follow-up, stratified by BMI group. In univariate Cox proportional hazards analyses, AIP as a continuous variable showed no significant association with the primary endpoint in patients with BMI ≥ 28 kg/m^2^ (HR 2.029; 95% CI 0.948–4.340; *p* = 0.068). In multivariate Cox proportional hazards analysis, the AIP only exhibited significant association with the primary endpoint in patients with BMI < 24 kg/m^2^ (HR 2.506; 95% CI 1.285–4.885; *p* = 0.007) and did not increase the risk for the primary endpoint in patients with BMI ≥ 24 kg/m^2^ (HR 1.747; 95% CI 0.921–3.316; *p* = 0.088 for patients with 24 kg/m^2^ ≤ BMI < 28 kg/m^2^; and HR 2.096; 95% CI 0.835–5.261; *p* = 0.115 for patients with BMI ≥ 28 kg/m^2^, respectively). Additionally, when the AIP was used as a continuous variable, it independently predicted the primary endpoint in the whole ACS patients (HR 2.000; 95% CI 1.344–2.976; *p* < 0.001).

**Figure 1:**
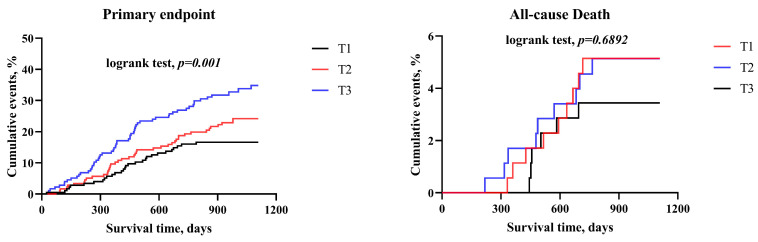

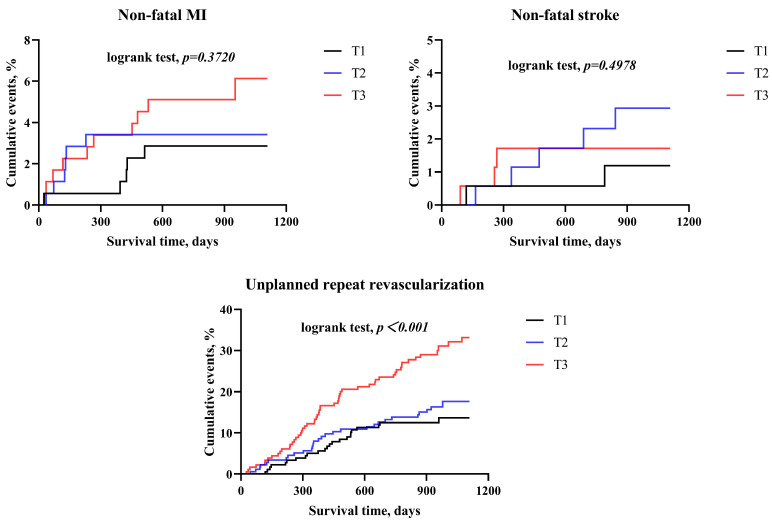
Kaplan–Meier curves for the primary and secondary endpoints based on AIP tertiles in the BMI < 24 kg/m^2^ group.

Figure 1 shows the Kaplan–Meier curves for the primary endpoint and secondary endpoints in different AIP tertiles within the BMI < 24 kg/m^2^ group. AIP as a continuous variable independently predicted unplanned repeat revascularization (HR 3.549; 95% CI, 1.798–7.005; *p* < 0.001).

Table 4 summarizes the findings from univariate and multivariate Cox proportional hazards analyses for the primary endpoint during follow-up within the BMI < 24 kg/m^2^ group. In the multivariate Cox proportional hazards analysis, using T1 as the reference, the AIP for T3 exhibited a significantly elevated hazard ratio (HR) for primary endpoint incidence (HR 1.772, 95% CI 1.110–2.828, *p* = 0.016).

## 4. Discussion

In the present study, we investigated the association between the AIP and the risk of adverse cardiovascular events in patients with ACS undergoing PCI, stratified by BMI levels. Our findings revealed no significant association between the AIP and adverse cardiovascular events in patients with a BMI ≥ 24 kg/m^2^. However, a significant correlation was observed between the AIP and cardiovascular event risk in patients with a BMI < 24 kg/m^2^. It is noteworthy that only five patients in our study had a BMI < 18.5 kg/m^2^, and our findings suggest that the AIP may serve as an independent risk factor for the ACS population, particularly among individuals with a seemingly healthy BMI. To the best of our knowledge, no prior studies have explored the impact of BMI on the prognostic significance of the AIP in ACS patients undergoing PCI. Our study adds to the growing body of evidence supporting the utility of the AIP as a predictive biomarker for adverse cardiovascular events, particularly in ACS patients, and highlights the significance of considering BMI as a potential modifier of this association.

Previous research has consistently highlighted the elevated risk of CAD in individuals with heightened levels of sdLDL compared to those with higher levels of large buoyant LDL (lbLDL) [25,26]. sdLDL is known to be more susceptible to oxidation and the formation of foam cells, rendering it a potential risk factor for atherosclerosis and cardiovascular events [27,28]. In 2002, the National Cholesterol Education Program (NCEP) recognized sdLDL as a major contributor to coronary heart disease (CHD) and recommended its assessment [29]. However, the practicality and costs associated with measuring sdLDL have constrained its routine use in clinical practice [9]. Nevertheless, the AIP, derived from a logarithmic transformation of the ratio of TG to HDL, demonstrates a strong correlation with LDL-C particle size and effectively reflects sdLDL levels [18,24]. The AIP has been established as a valuable tool for assessing the risk of adverse cardiovascular events in diverse populations [14,15,16,17,18,19,20,21]. This underscores its potential as a more feasible alternative to measuring sdLDL for risk stratification in clinical practice.

The results of a cohort study revealed that ACS patients exhibited a significant increase in sdLDL serum concentrations compared to the healthy control group. Additionally, a Spearman correlation analysis has demonstrated a statistically significant, albeit weak, positive correlation between sdLDL levels and the AIP; r = 0.32 (*p* < 0.001) [30]. Previous studies have underscored the prognostic significance of the AIP in predicting adverse cardiovascular events in individuals afflicted with CAD. A meta-analysis encompassing data from ten studies exploring the relationship between the AIP and cardiovascular events has indicated that an elevated AIP value may be independently linked to CAD within the adult population [18]. Furthermore, Cai G et al. have conducted a study revealing that the AIP exhibits an independent association with the presence and severity of ACS, with the association manifesting in a sex-dependent manner. As the AIP tertiles increased, there was a corresponding elevation in the prevalence of ACS, acute MI, unstable angina pectoris, and the Gensini score (a scoring system employed for assessing the severity of CAD) [19]. Ma et al. have also reported that an increased AIP value independently and strongly correlates with adverse cardiovascular events in patients with type 2 diabetes mellitus (T2DM) who have ACS and are undergoing PCI [20]. Zheng et al. have highlighted the utility of the AIP index as a prognostic tool for non-diabetic CAD patients two years post-PCI. A J-shaped restricted cubic spline (RCS) curve accentuated a change in the HR after the 0.18 juncture (HR 1.20, 95% CI 0.96–1.50) [21]. Y. ÖZEN et al. retrospectively analyzed the data of 1154 ACS patients between January 2017 and December 2018.Their investigation revealed that the AIP serves as an independent risk factor for major adverse cardiovascular events (MACE) within the ACS population [16]. However, BMI levels were not grouped in detail in those studies. In our current study, we have ascertained a significant association between the AIP and an increased risk of adverse cardiovascular events, specifically in ACS patients with a BMI less than 24 kg/m^2^. Importantly, the predominant form of cardiovascular adverse events observed in this subgroup appears to be revascularization, consistent with previous research findings [20,21].

Interestingly, in the present study, the AIP only has prognostic value for adverse cardiovascular events in ACS people with BMI < 24 kg/m^2^ (HR 2.506; 95% CI 1.285–4.885; *p* = 0.007). In groups of 24 kg/m^2^ ≤ BMI < 28 kg/m^2^ and BMI > 28 kg/m^2^, the AIP does not show prognostic value for adverse cardiovascular events. It is worth mentioning that the AIP as a continuous variable is significantly associated with adverse cardiovascular events in our whole ACS population (HR 2.000; 95% CI 1.344–2.976; *p* < 0.001). Recently, one study demonstrated that the AIP could predict the incidence of stroke only in people with a healthy BMI [17]. In addition to adverse cardiovascular events, the impact of BMI on the prognostic value of the AIP has also been observed in other studies. Li et al. found that the association of the TG/HDL-C ratio with hyperinsulinemia was stronger among people with a BMI < 25 kg/m^2^ than those with BMI ≥ 30 kg/m^2^ [31]. An observational study showed that the discriminatory power of TG/HDL-C for insulin resistance was acceptable in women with BMI < 24 kg/m^2^ (AROCs: 0.718) and was not acceptable in those with BMI ≥ 24 kg/m^2^ [32]. The results of these studies all indicate that for individuals with a relatively low BMI, the AIP has a higher prognostic value. Thus, it is worth further exploring whether the prognostic value of the AIP is consistent among different BMI populations. It is not clear whether our results can be extended to different populations and whether the prediction of other endpoints such as DM and metabolic dysfunction is affected by BMI. Various populations and larger sample sizes are needed to verify this association in further study.

Several limitations exist in the present study. Firstly, the small sample size of only five patients with a low body weight may limit the generalizability of our findings to the overall population with a BMI below 24 kg/m^2^. Instead, our sample may be more representative of the normal body weight population. Secondly, the baseline concentrations of triglycerides and HDL-C could have been influenced by the use of lipid-lowering drugs, although no significant differences were observed among participants regarding the use of statins. Additionally, in our study, LDL-C levels were calculated using the Friedewald equation. However, at the low levels targeted in individuals with coronary artery disease, LDL-C values may not be very accurate. Thirdly, unhealthy lifestyle factors and obstructive sleep apnea are known to be associated with dyslipidemia and the development of cardiovascular disease. Unfortunately, this study did not record or consider information about lifestyle factors such as diet and exercise, as well as obstructive sleep apnea. Fourthly, since the ratio of women is higher in BMI < 24 kg/m^2^ group, the effect of BMI described in our results may be a bit exaggerated by the increased proportion of females in the group below 24 kg/m^2^. However, we have already modified cardiovascular risk factors including gender ratio to minimize this influence. Lastly, while the ethnic homogeneity of the study population can be seen as an advantage, caution should be exercised in extrapolating the findings to other ethnic groups.

## 5. Conclusions

In conclusion, our results demonstrate that an increased AIP correlated significantly with adverse cardiovascular events risk only in ACS patients undergoing PCI with BMI < 24 kg/m^2^, but not in patients with BMI ≥ 24 kg/m^2^. Measurement of the AIP may help to predict adverse cardiovascular events in ACS patients with BMI < 24 kg/m^2^. Whether this relationship exists in other populations needs to be confirmed in further study.

## Figures and Tables

**Table 1:** Baseline characteristics of the patients.

Variable	BMI < 24 kg/m^2^					24 kg/m^2^ ≤ BMI < 28 kg/m^2^	BMI > 28 kg/m^2^
	Total	T1	T2	T3	*p* Value		
	N = 526	N = 175	N = 176	N = 175		N = 827	N = 372
**Demographics**							
Age (years)	62 ± 10	63 ± 9	61 ± 9	60 ± 12	0.013 *	60 ± 10	57 ± 11
Male sex, n (%)	338 (64.3)	106 (60.6)	118 (67.0)	114 (65.1)	0.429	676 (81.7)	309 (83.1)
**Risk factors**							
Current smoking, n (%)	193 (36.7)	55 (31.4)	62 (35.2)	76 (43.4)	0.059	388 (46.9)	182 (48.9)
Chronically daily drinking, n (%)	53 (10.1)	20 (11.4)	9 (5.1)	24 (13.7)	0.021 *	121 (14.6)	56 (15.1)
Family history of CHD, n (%)	161 (30.7)	47 (26.9)	57 (32.4)	57 (32.6)	0.419	259 (31.4)	130 (34.8)
Hypertension, n (%)	302 (57.4)	104 (59.4)	101 (57.4)	97 (55.4)	0.751	528 (63.8)	269 (72.3)
Dyslipidemia, n (%)	379 (72.1)	75 (42.9)	134 (76.1)	170 (97.1)	<0.001 *	678 (82.0)	323 (86.8)
Diabetes, n (%)	219 (41.6)	57 (32.6)	89 (50.6)	73 (41.7)	0.003 *	395 (47.8)	181 (48.7)
PAD, n (%)	60 (11.4)	15 (8.6)	29 (16.5)	16 (9.1)	0.034 *	79 (9.6)	38 (10.2)
Cardiac failure, n (%)	45 (8.6)	10 (5.7)	19 (10.8)	16 (9.1)	0.222	57 (6.9)	18 (4.8)
CKD, n (%)	57 (10.8)	20 (11.4)	15 (8.5)	22 (12.6)	0.453	34 (4.1)	9 (2.4)
Previous MI, n (%)	101 (19.2)	33 (18.9)	34 (19.3)	34 (19.4)	0.990	145 (17.5)	85 (22.8)
Past PCI, n (%)	102 (19.4)	40 (22.9)	28 (15.9)	34 (19.4)	0.258	161 (19.5)	79 (21.2)
LVEF (%)	65 (60–68)	65 (61–69)	64.5 (60–68)	64 (59–68)	0.143	64 (60–68)	64 (59–68)
**Clinical presentation**							
UA, n (%)	382 (72.6)	152 (86.9)	126 (71.6)	104 (59.4)	<0.001 *	622 (75.2)	277 (74.5)
NSTEMI, n (%)	74 (14.1)	17 (9.7)	21 (11.9)	36 (20.6)	0.009 *	95 (11.5)	50 (13.4)
STEMI, n (%)	70 (13.3)	6 (3.4)	29 (16.5)	35 (20.0)	<0.001 *	110 (13.3)	45 (12.1)
**Laboratory measurements (fasting state)**							
TC (mmol/L)	4.15 ± 0.95	4.00 ± 0.90	4.08 ± 0.92	4.36 ± 0.98	0.001 *	4.09 ± 0.99	4.27 ± 1.03
TG (mmol/L)	1.31 (0.91–1.86)	0.81 ± 0.22	1.31 ± 0.29	2.42 ± 1.01	<0.001 *	1.44 (1.02–2.06)	1.70 (1.23–2.48)
LDL-C (mmol/L)	2.42 ± 0.79	2.23 ± 0.78	2.46 ± 0.77	2.56 ± 0.79	<0.001 *	2.40 ± 0.80	2.56 ± 0.83
HDL-C (mmol/L)	1.09 ± 0.26	1.30 ± 0.24	1.05 ± 0.18	0.92 ± 0.18	<0.001 *	1.02 ± 0.22	0.97 ± 0.21
FPG (mmol/L)	5.67 (5.18–6.72)	5.54 (5.08–6.21)	5.80 (5.26–7.18)	5.78 (5.18–6.83)	0.002 *	5.87 (5.23–7.08)	5.88 (5.27–7.01)
Glycated hemoglobin (%)	6.0 (5.5–7.1)	5.8 (5.5–6.6)	6.3 (5.6–7.6)	6.0 (5.6–7.1)	0.004 *	6.1 (5.6–7.2)	6.2 (5.6–7.0)
Hs-CRP (mg/L)	1.32 (0.61–3.13)	0.94 (0.39–2.18)	1.29 (0.61–2.89)	1.84 (0.81–4.97)	<0.001 *	1.30 (0.61–3.39)	1.71 (0.73–4.31)

* A significance level of *p* < 0.05 was considered statistically significant. CHD Coronary heart disease; CKD Chronic kidney disease; PAD Peripheral artery disease; TC Total cholesterol; TG triglycerides.

**Table 2:** Use of angiographic findings, procedural results, and medications.

Variable	BMI < 24 kg/m^2^					24 kg/m^2^ ≤ BMI < 28 kg/m^2^	BMI > 28 kg/m^2^
	Total	T1	T2	T3	*p* Value		
	N = 526	N = 175	N = 176	N = 175		N = 827	N = 372
**Medications at discharge**							
Aspirin, n (%)	516 (98.1)	171 (97.7)	173 (98.3)	172 (98.3)	0.901	823 (99.5)	370 (99.5)
P2Y12, n (%)	525 (99.8)	175 (100.0)	175 (99.4)	175 (100.0)	0.369	826 (99.9)	372 (100.0)
Statins, n (%)	526 (100.0)	175 (100.0)	176 (100.0)	175 (100.0)	>0.999	827 (100.0)	372 (100.0)
ACEIs/ARBs, n (%)	231 (43.9)	71 (40.6)	84 (47.7)	76 (43.4)	0.397	403 (48.7)	197 (53.0)
β-blockers, n (%)	356 (67.7)	111 (63.4)	131 (74.4)	114 (65.1)	0.06	588 (71.1)	267 (71.8)
**Angiographic findings**							
One-vessel disease, n (%)	94 (17.9)	37 (21.1)	28 (15.9)	29 (16.6)	0.379	117 (14.1)	51 (13.7)
Two-vessel disease, n (%)	145 (27.6)	51 (29.1)	42 (23.9)	52 (29.7)	0.4	232 (28.1)	111 (29.8)
LM/three-vessel disease, n (%)	287 (54.6)	87 (49.7)	106 (60.2)	94 (53.7)	0.136	478 (57.8)	210 (56.5)
Restenotic lesions, n (%)	67 (12.7)	25 (14.3)	16 (9.1)	26 (14.9)	0.203	94 (11.4)	41 (11.0)
Chronic total occlusions, n (%)	107 (20.3)	35 (20.0)	33 (18.8)	39 (22.3)	0.706	171 (20.7)	87 (23.4)
Syntax score	21.59 ± 11.62	19.98 ± 11.04	22.63 ± 12.21	21.59 ± 11.62	0.074	21.23 ± 11.90	20.78 ± 9.90
**Procedural results**							
DCB	31 (5.9)	12 (6.9)	6 (3.4)	13 (7.4)	0.224	48 (5.8)	32 (8.6)
DES	429 (81.6)	142 (81.1)	152 (86.4)	135 (77.1)	0.082	693 (83.8)	294 (79.0)
BRS	36 (6.8)	13 (7.4)	9 (5.1)	14 (8.0)	0.525	45 (5.4)	17 (4.6)
Complete revascularization, n (%)	317 (60.3)	117 (66.9)	99 (56.3)	101 (57.7)	0.089	529 (64.0)	213 (57.3)

ARBs Angiotensin II receptor blockers; ACEIs Angiotensin converting enzyme inhibitors; LM Left-main artery; DES Drug-eluting stent; DCB Drug-coated balloon; BRS Bioresorbable scaffold.

**Table 3:** Univariate and multivariate Cox proportional hazards analyses for the primary endpoint between different BMI groups.

Variables	Univariate Analysis						Multivariate Analysis					
	BMI < 24 kg/m^2^		24 kg/m^2^ ≤ BMI < 28 kg/m^2^		BMI ≥ 28 kg/m^2^		BMI < 24 kg/m^2^		24 kg/m^2^ ≤ BMI < 28 kg/m^2^		BMI ≥ 28 kg/m^2^	
	HR	*p*-Value	HR	*p*-Value	HR	*p*-Value	HR	*p*-Value	HR	*p*-Value	HR	*p*-Value
AIP	3.125	<0.001 *	2.431	0.002 *	2.029	0.068	2.506	0.007 *	1.747	0.088	2.096	0.115
Male sex	1.029	0.877	1.377	0.219	0.927	0.791	0.819	0.378	1.289	0.356	0.662	0.221
Age (years)	1.006	0.523	1.013	0.144	1.002	0.859	0.987	0.200	0.989	0.296	0.999	0.942
Current smoking	1.392	0.063	1.098	0.577	1.087	0.7	1.541	0.047 *	0.929	0.707	1.049	0.852
Hypertension	1.136	0.481	1.06	0.741	0.948	0.822	1.266	0.217	1.023	0.902	1.042	0.875
Diabetes	1.386	0.065	2.369	<0.001 *	0.916	0.688	1.313	0.142	1.981	<0.001 *	0.826	0.411
NSTE-ACS	1.332	0.312	1.017	0.944	0.577	0.053	2.094	0.028 *	1.003	0.990	0.703	0.287
Cardiac failure	1.502	0.139	2.373	<0.001 *	1.764	0.15	0.965	0.907	1.684	0.053	2.174	0.081
CKD	1.877	0.008 *	3.724	<0.001 *	0.472	0.455	1.627	0.089	2.324	0.008 *	0.355	0.321
Previous MI	1.894	<0.001 *	1.487	0.043 *	1.093	0.725	1.294	0.324	0.956	0.838	0.730	0.330
Previous PCI	1.497	0.046 *	1.752	0.002 *	1.335	0.244	1.391	0.200	1.699	0.012 *	1.495	0.184
LDL-C	1.406	<0.001 *	0.992	0.941	1.187	0.158	1.252	0.048 *	1.047	0.678	1.214	0.159
Hs-CRP	1.059	<0.001 *	1.03	0.014 *	0.998	0.904	1.061	<0.001 *	1.018	0.211	0.966	0.141
**STNTAX Score**												
Score ≤ 22	Ref		Ref		Ref		Ref		Ref		Ref	
22 < Score < 33	1.387	0.119	2.299	<0.001 *	1.553	0.074	0.978	0.925	1.463	0.070	1.439	0.171
Score ≥ 33	2.292	<0.001 *	1.98	0.003 *	2.974	<0.001 *	1.396	0.207	1.250	0.387	2.582	0.004 *
Complete revascularization	0.631	0.009 *	0.352	<0.001 *	0.376	<0.001 *	0.729	0.118	0.444	<0.001 *	0.422	0.001 *

* A significance level of *p* < 0.05 was considered statistically significant.

**Table 4:** Univariate and multivariate Cox proportional hazards analyses for the primary endpoint within the BMI < 24 kg/m^2^ group.

Variables	Univariate Analysis		Multivariate Analysis	
	HR	*p*-Value	HR	*p*-Value
**AIP tertiles**				
T1	Reference		Reference	
T2	1.448 (0.900–2.329)	0.127	1.201 (0.729–1.980)	0.472
T3	2.216 (1.419–3.460)	<0.001 *	1.772 (1.110–2.828)	0.016 *
Male sex	1.029 (0.715–1.482)	0.877	0.820 (0.525–1.280)	0.382
Age (years)	1.006 (0.989–1.023)	0.523	0.986 (0.967–1.006)	0.160
Current smoking	1.392 (0.982–1.974)	0.063	1.577 (1.026–2.424)	0.038
Hypertension	1.136 (0.797–1.618)	0.481	1.273 (0.877–1.849)	0.205
Diabetes	1.386 (0.980–1.961)	0.065	1.339 (0.926–1.935)	0.121
NSTEACS	1.332 (0.764–2.321)	0.312	2.076 (1.074–4.010)	0.030 *
Cardiac failure	1.502 (0.876–2.574)	0.139	0.973 (0.533–1.778)	0.929
CKD	1.502 (1.176–2.998)	0.139	1.672 (0.953–2.934)	0.073
Previous MI	1.877 (1.296–2.768)	0.008	1.318 (0.791–2.195)	0.289
Previous PCI	1.894 (1.007–2.224)	<0.001 *	1.384 (0.835–2.295)	0.208
LDL-C	1.497 (1.150–1.720)	0.046 *	1.239 (0.991–1.550)	0.060
Hs-CRP	1.406 (1.037–1.082)	<0.001 *	1.060 (1.033–1.088)	<0.001 *
**STNTAX Score**				
Score ≤ 22	Reference		Reference	
22 < Score < 33	1.387 (0.920–2.092)	0.119	1.011 (0.641–1.597)	0.961
Score ≥ 33	2.292 (1.490–3.525)	<0.001 *	1.402 (0.832–2.362)	0.204
Complete revascularization	0.631 (0.446–0.892)	0.009 *	0.739 (0.496–1.101)	0.137

* *p* < 0.05 is considered statistically significant.

## Data Availability

All data generated or analyzed during this study are included in this published article.
